# Segmentation of Multi-Isotope Imaging Mass Spectrometry Data for Semi-Automatic Detection of Regions of Interest

**DOI:** 10.1371/journal.pone.0030576

**Published:** 2012-02-09

**Authors:** Philipp Gormanns, Stefan Reckow, J. Collin Poczatek, Christoph W. Turck, Claude Lechene

**Affiliations:** 1 Max Planck Institute of Psychiatry, Proteomics and Biomarkers, Munich, Germany; 2 National Resource for Imaging Mass Spectrometry, Harvard Medical School, and Brigham and Women's Hospital, Cambridge, Massachusetts, United States of America; University of Glasgow, United Kingdom

## Abstract

Multi-isotope imaging mass spectrometry (MIMS) associates secondary ion mass spectrometry (SIMS) with detection of several atomic masses, the use of stable isotopes as labels, and affiliated quantitative image-analysis software. By associating image and measure, MIMS allows one to obtain quantitative information about biological processes in sub-cellular domains. MIMS can be applied to a wide range of biomedical problems, in particular metabolism and cell fate [1], [2], [3]. In order to obtain morphologically pertinent data from MIMS images, we have to define regions of interest (ROIs). ROIs are drawn by hand, a tedious and time-consuming process. We have developed and successfully applied a support vector machine (SVM) for segmentation of MIMS images that allows fast, semi-automatic boundary detection of regions of interests. Using the SVM, high-quality ROIs (as compared to an expert's manual delineation) were obtained for 2 types of images derived from unrelated data sets. This automation simplifies, accelerates and improves the post-processing analysis of MIMS images. This approach has been integrated into “Open MIMS,” an ImageJ-plugin for comprehensive analysis of MIMS images that is available online at http://www.nrims.hms.harvard.edu/NRIMS_ImageJ.php.

## Introduction

We developed software to automatically segment quantitative images obtained with multi-isotope mass spectrometry (MIMS). To understand the turnover of proteins, we developed MIMS, a method that could reveal new protein synthesis with high spatial resolution, in adult animals, in vivo, and without transfection of cells or over-expression of protein. We provide mice with a diet enriched with the stable isotope ^15^N, which has a low natural abundance. Newly synthesized proteins would therefore contain ^15^N in nearly the same proportion as in the enriched food. To detect the location of ^15^N-tagged protein, we fix and section tissues as for histology, and we use MIMS to measure the ratio of ^15^N to ^14^N at each location in a field ranging from a few to 100 µm. Because each pixel in an image is created from counts of specific atomic masses, the extent of ^15^N incorporation can be measured with exceptional spatial resolution and a precision that depends only on the time allowed to acquire an image. Because MIMS is based on stable isotopes, it is applicable to human studies.

MIMS combines a new type of secondary ion mass spectrometer, the use of stable isotope reporters, and intensive computation. Secondary Ion Mass Spectrometry is based upon the sputtering of a few atomic layers from the surface of a sample, induced by a ‘primary ion’ bombardment. Images are obtained by stepping the primary ion beam across the sample. For each step location on the sample, the number of secondary ions sputtered is recorded. MIMS images represent the variation in intensity of each selected secondary ion species across the pixels of the area scanned. We locate and measure the experimentally induced enrichment of a specific stable isotope in a sample by deriving a ratio image from the pixel-wise division of individual masses (e.g. ratio ^12^C^15^N^−^/^12^C^14^N^−^). For further discussion of the instrumentation and methods described see [1], [2].

**Figure 1:**
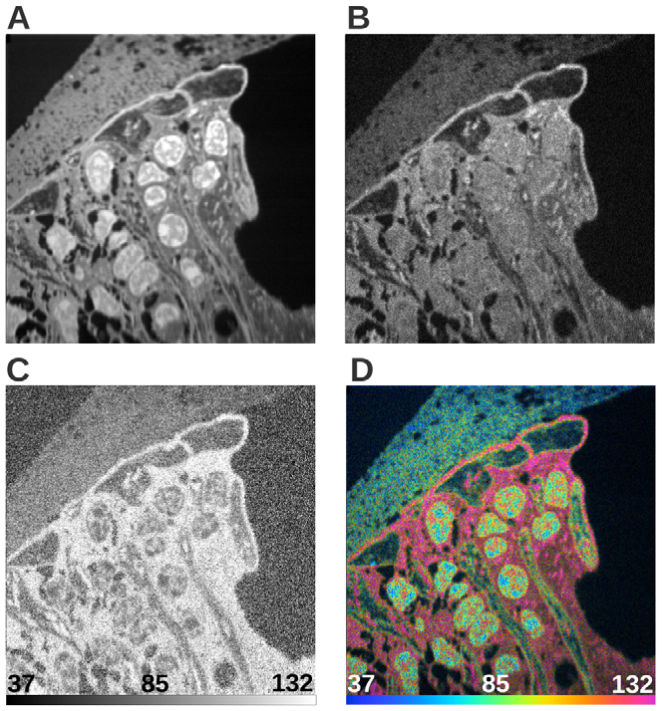
MIMS images of mouse cochlea tissue sections. Quantitative images based on measured masses of ^12^C^14^N^−^ (A) and ^12^C^15^N^−^ (B), representing the detected amount of the respective nitrogen isotopes at each pixel. Dividing the values at each pixel results in a ratio image (C), which determines the isotopic ratio of nitrogen at each position within the section. (D) is the ^12^C^15^N/^12^C^14^N HSI of the data. Scale bars on (C) and (D) range from the natural ratio to the value in the ^15^N-enriched chow, which corresponds to the maximum ratio that could be reached in newly synthesized protein (multiplied by 10000). Field 41×41 µm, 512×512 pixels, acquisition time 240 minutes.

A critical step in the MIMS image analysis is the definition of regions of interest (ROIs)—groups of neighboring pixels exhibiting a distinct set of features that distinguish them from the surrounding area—from which statistics are collected and interpreted. Images acquired with MIMS have a dynamic range of 16 bits, and the resulting ratio images generate far more information than can be easily displayed using simple gray or color level methods. In order to show this high dynamic range ratio data, and to de-emphasize values resulting from data with few counts, we have developed a method based on a hue saturation intensity transformation (HSI) [1], [2] of the ratio image. The hue codes for the ratio value, and the intensity at a given hue codes for the number of ions detected. The HSI ratio display allows us to take full advantage of our increased perception of color and of the quantitative information contained in each mass image. It enables identification of ROIs with significant excess of the ^15^N label (or any other isotopic label) by a means that is independent of visual recognition of expected histological structures. Figure 1A–C shows a MIMS image (^12^C^14^N and ^12^C^15^N mass images, and the ^12^C^15^N/^12^C^14^N ratio image) of a tissue section from the inner ear of a mouse (specifically the cochlea), and Figure 1D shows the HSI of the ^12^C^15^N/^12^C^14^N ratio of that same image. Yet, even with the HSI image as a guide, the manual delineation of ROIs is extremely time consuming and rarely encompasses the information contained in an entire image.

Here we describe the automatic derivation of MIMS image ROIs by segmentation after algorithm training. Indeed, detecting ROIs is not unlike image segmentation, where the goal is the partitioning of an image into non-overlapping, constituent regions that are homogeneous with respect to some characteristic. Common image segmentation methods range from simple thresholding to sophisticated approaches of edge and surface detection [4]. Segmentation is particularly challenging in the medical imaging domain, since data generated from magnetic resonance imaging (MRI) or computed tomography (CT) are inherently complex and noisy. Machine learning methods have been used successfully to tackle these issues [4], [5].

To segment MIMS images, we have utilized a Support Vector Machine (SVM) [6], [7], [8], [9]. SVMs have been applied to numerous and highly varied types of biological data, of which some examples are: microarray gene expression data for both gene function classification [10] and cancer classification [11], protein secondary structure prediction [12], detection of microcalcifications in mammograms [13], and proteomic time-of-flight mass spectrometry data [14]. Classification using an SVM involves training a model on a defined set of data in which each data point is represented in an experiment-dependent feature space and assigned to a certain class. If there are *d* measured parameters, the ranges of these parameters create a space of *d* dimensions containing the measured data points. For any one of these parameters, the range of values can be partitioned so that each component of the partition represents a class. Briefly, in an SVM a space of dimension *d* is partitioned by *d-1* dimensional hyperplanes denoting different classes of data points. Here a *hyperplane* is the higher dimensional analog to a plane partitioning a 3 dimensional space into 2 classes. A short discussion of SVMs can be found in (Text S1, Figure S1, Figure S2). More in-depth descriptions can be found in [15] and [16], and an overview of SVMs applied to biological data can be found in [17].

The model can be subsequently applied to data points of unknown class in order to infer the class membership of each data point. The classification of each pixel in a MIMS image can be regarded as a full segmentation, and local clusters of pixels of the same class represent potential regions of interest. We present results from the application of the method to two different types of MIMS images and investigate its usefulness for streamlining data analysis.

## Results

### Experimental Data

**Figure 2:**
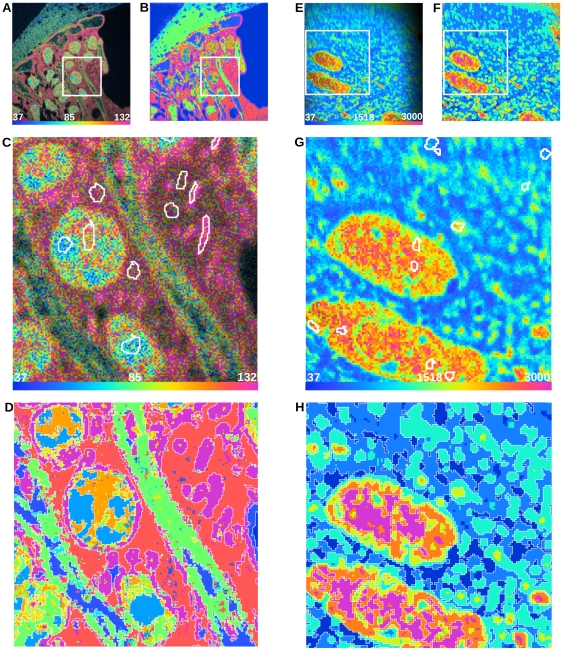
HSI images, Expert ROIs, and Segmentation Results. (A) is the ^12^C^15^N/^12^C^14^N HSI image of a mouse cochlea section with a field of 41×41 µm, 512×512 pixels, and acquisition time of 240 minutes. Scale bars range from the natural ratio to the value in the slightly ^15^N-enriched chow, which corresponds to the maximum ratio that could be reached in newly synthesized protein (multiplied by 10000). (B) is a complete segmentation of (A). (C) and (D) are insets of (A) and (B), respectively. (C) shows expert freehand-drawn ROIs (white borders) used for training the SVM. (D) is the complete segmentation of (C). We chose 6 classes of ratio value ensembles guided by the HSI image and represented in (B) and (D) by 6 hues spanning the rainbow colors from blue (lowest ratio) to red (highest). The SVM used the ^12^C^14^N quantitative MIMS image and the derived ^12^C^15^N/^12^C^14^N ratio image. (E) is the ^12^C^15^N/^12^C^14^N HSI image of a mouse brain section, field 50×50 µm, 256×256 pixels, acquisition time of 11 minutes. Scale bars range from the natural ratio to the maximum value measured in the brain after feeding with a maximally 15N-enriched chow (∼98%). (F) is a complete segmentation of (E). (G) and (H) are insets of (E) and (F), respectively. (G) shows expert freehand-drawn ROIs (white borders) used for training the SVM. We chose 6 classes of ratio value ensembles guided by the HSI image and represented in (F) and (H) by 6 hues spanning the rainbow colors from blue (lowest ratio) to red (highest). The SVM used only the derived ^12^C^15^N/^12^C^14^N ratio image. ROI boundaries (which have zero width) in (C) and (G) have been thickened to 1 pixel for clarity.

We present results obtained using our algorithm to segment quantitative MIMS images of mouse cochlea and brain. These images were generated in studies of protein turnover after incorporation of ^15^N, which was provided in the mouse chow. Cochlea images (Figures 1 and 2A–D) are from a study of protein turnover using a diet slightly enriched with ^15^N-leucine [1]. Figure 2B is the complete segmentation result of 2A. Insets marked in 2A and 2B are shown in 2C and 2D, respectively. Regions of interest have been defined by an expert to denote different classes within the images and are visible in Figure 2C (white borders). All pixels within the ROIs are used as ground-truth example data points for their respective class. Figure 2D (inset from 2B) shows the segmentation result in detail with the boundaries of ROIs highlighted in white. Brain images (Figures 2E–H) are from a study with highly ^15^N enriched spirulina chow (isotope enrichment >98%) [18]. Figure 2E shows the ^12^C^15^N/^12^C^14^N HSI from a tissue section of the hippocampal region of a mouse brain. Figure 2F is the complete segmentation result of 2E. Insets marked in 2E and 2F are shown in 2G and 2H, respectively. The expert-defined ROIs are visible in 2G (white borders), and the segmentation result is seen in detail with the boundaries of ROIs highlighted in white in Figure 2H. A figure detailing the acquisition of the brain images may be found in (Figure S4).

The validation of image segmentation by the algorithm must take into account several characteristics specific to MIMS. Expert analysis should sample various regions of the image that correspond to specific biological structures, either directed by the histology of the image (e.g., Figure 1A) or by the level of label incorporation (e.g., Figure 2A, E). While an expert had previously defined classes and training data for both MIMS images, the amount of expert-annotated data points varies between classes as well as between images.

### Feature Space and SVM-Based Segmentation

The data points to be classified by the SVM are the pixels in the MIMS image. Here, “pixel” refers to a specific position within the raster used to image the sample. Since MIMS measures multiple isotopic masses at each position, each pixel is in fact represented by a set of intensities (one for each measured mass). Also, since the SVM is able to use information from all measured masses at the same time, the classification is not restricted to any single mass or ratio image, but can be applied to all or part of the of the MIMS data, here simply referred to as “MIMS image.” It should be stated, however, that the inclusion of all mass or ratio images is neither required nor necessarily desirable. Explicitly in the algorithm evaluations that follow in the case of the cochlea data, both the ^12^C^14^N mass image (Figure 1A) and the ^12^C^15^N/^12^C^14^N ratio image (Figure 1C) are given to the SVM. In the case of the brain data, only ^12^C^15^N/^12^C^14^N ratio images (as represented in the HSI images presented in Figure 2E) are used for reasons explained below in the section “*Cross Segmentation*”. Each data point in a MIMS image is described by the following set of features: the pixel intensity value, the mean of intensity values of neighboring pixels, the standard deviation of this mean, and finally the magnitude of intensity value gradient in this neighborhood.

The intensity value is an estimate of the abundance of an atomic mass within the sample at the pixel's location, and the direct result of the measurement. Because we are interested in areas of homogeneous relative intensities, we consider a local intensity distribution with respect to each pixel's neighborhood. In addition, MIMS measurements can be noisy due to low ion counts, specifically low-count mass images and ratio images where the noise of the lower-count mass dominates. In order to make up for sampling errors in a single pixel's intensity, neighboring pixels are taken into account during classification. Both the mean and standard deviation of intensities of adjacent pixels are calculated as two additional features for the SVM. The size of this neighborhood can be increased, for example to accommodate varying levels of noise between measurements. The distributional information, however, might be misleading at the border of segments where neighboring pixels belong to different classes. In order to detect these boundaries, we also include an approximated gradient magnitude calculation for each pixel as a fourth feature.

Overall, the SVM and the testing account for the following: 1) all four features (pixel value, as well as neighborhood mean, standard deviation, and gradient magnitude) are derived from each of the mass and/or ratio images individually without positional information in the feature space; 2) the desired segmentation is multi-class; 3) there is no complete expert segmentation; 4) the underlying distribution of protein turnover (in the case of both the cochlea and brain images) is unknown and is comprised of an unknown number of sub-populations; 5) the expert decides how many classes exist in the data. The first two facts result in a rich feature space, integrating information from multiple isotopic species in order to differentiate between classes in the complex MIMS data set. The use of an SVM and of this feature space is based on the work of Zawadzki et al. [19].

During classification, each data point is assigned to a class based on its feature representation. A classification of all pixels in a MIMS image represents a full segmentation. One segment is defined as a set of connected pixels belonging to the same class. Derivation of regions of interest is straightforward by tracing the borders around connected pixels that are assigned to the same class. If the size of an ROI of a given class falls below a user-defined number of pixels, it is integrated into its surrounding segment. Furthermore, if there are segments of sufficient size completely contained within another segment, their area is subtracted from the surrounding segment. The user can control this behavior by varying the size threshold, and the subtraction guarantees that a pixel can never be assigned to more than one ROI.

### Performance Metrics

To assess the predictive performance of our SVM approach, we investigated its ability to reproduce results generated by the expert sampling the image via manual selection of ROIs. We compared predicted class memberships of individual pixels to this expert-selected class assignment. To account for possible variation in performance between different classes, we assessed each class individually by calculating “recall” and “precision.” Recall of class “C” is the percentage of pixels correctly classified. It is calculated by the number of pixels correctly classified by the SVM as “C” (belonging to class C defined by the expert) divided by the total number of pixels belonging to class “C” as defined by the expert, giving the equation: recall = N^*^_C_/T_C_. Here N^*^_C_ denotes the number of pixels correctly classified as “C” by the SVM and *T_C_* the total number of pixels belonging to class C as assigned by the expert. Precision of class “C” is calculated by the number of pixels correctly classified as “C” divided by the total number of pixels classified as “C” (correct and incorrect assignments of the algorithm), giving: precision = N^*^_C_/(N^*^_C_+N°_C_). Here *N°_C_* denotes the number of pixels incorrectly classified as “C”. Both metrics are given as percentages in the following paragraphs. When assessing total performance, an evaluation based on both the recall and precision for each class avoids introducing bias due to unequal class sizes, which is typical for most MIMS data sets.

### Random Sampling

We used a random sub-sampling validation scheme to evaluate the performance on different data subsets. The expert-selected data were randomly split into two fractions of defined sizes; one group was used to train the classifier and the other to test it. The size of the fractions was varied to investigate the effect of training data size on the recall and precision. The relative amounts of training data between classes were kept constant. Sampling was done in one of two ways: *ROI sampling* was based on complete expert-drawn ROIs, where all pixels within the ROI were either in a training or test set; and *pixel sampling*, in which pixels from all expert drawn ROIs of the same class were combined and individual pixels were randomly sampled from the whole set.

### Algorithm Evaluation

#### Predictive Performance: Recall and Precision

The expert-annotated data points were randomly assigned to training and test sets 500 times. To perform the large number of training iterations efficiently, SVM parameter optimization was performed by means of the Nelder-Mead heuristic [20] (Text S1). Here *heuristic* denotes an algorithm that is not guaranteed to find the global optima. Although this algorithm is prone to getting stuck in local optima resulting in suboptimal classification, the performance of the approach is well approximated by the average values of recall and precision after many iterations. Recall and precision were calculated for each iteration, and the probability density function was visualized using violin plots (Text S1, Figure S3). Violin plots were used in place of box and whisker plots due to the highly non-normal distributions of recall and precision.

**Figure 3:**
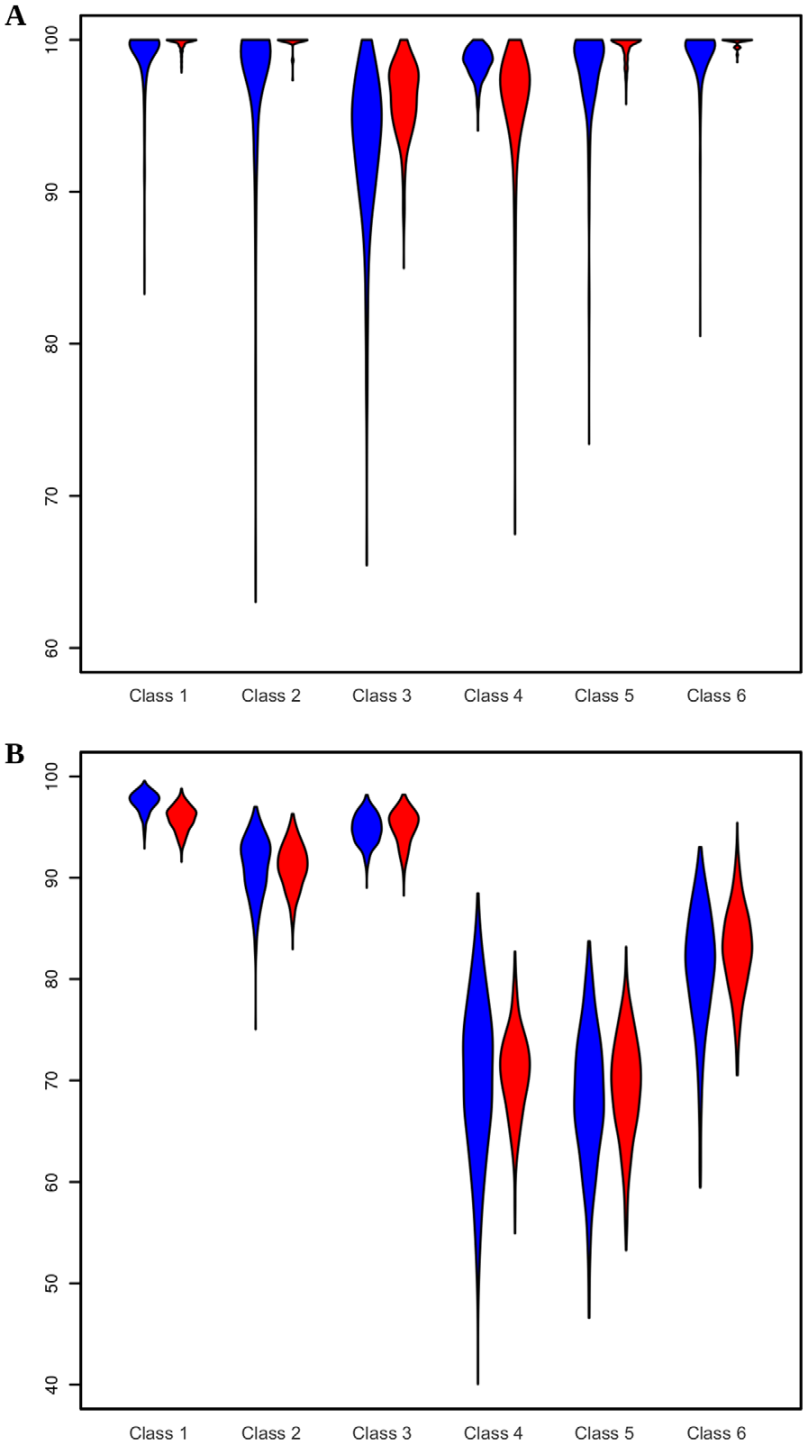
Cross-validation results of performance analysis. Violin plots showing the probability density functions of classification performance on MIMS images of the cochlea (A) and brain (B), Figures 2A and 2E, respectively. For each class, recall (blue) and precision (red) were calculated by cross-validation on 20% of the expert-annotated ROIs. N = 500 in both cases.

We achieve high recall and precision values on the cochlea image (Figure 2A) using pixel sampling (20% of annotated pixels in training set, randomly sampled from all expert selected pixels as described above). As illustrated in Figure 3A, all classes show average recall and precision greater than 90%, demonstrating excellent classification performance across all user defined classes. In case of the brain image (Figure 2E), the number of expert-drawn ROIs was much larger than in the case of the cochlea image; an ROI sampling scheme was applied (20% of annotated ROIs in the training set, randomly sampled from all expert selected ROIs as described above), resulting in stable recall and precision values for most classes as shown in Figure 3B. On average, four out of six classes have recall and precision above 80%. Only classes 4 and 5 show impaired performance, with average recall and precision of about 70% and a considerably higher variance. Closer inspection of the expert-annotated data revealed a significantly higher feature similarity between those classes than between others (data not shown). Additionally, the total number of pixels defining these two classes was lower than for other classes. Both factors might well account for the reduced classification accuracy. In summary, the SVM performs well on the MIMS data, but the results demonstrate the need for careful classification for a successful segmentation of all classes. It is also apparent that the probability density functions of both precision and recall are highly non-normal. The long lower tails are due to the fact that there is a small probability of the Nelder-Mead algorithm (being a directed random search) finding parameters far from the global maximum in terms of accuracy.

#### Robustness Analysis

The algorithm is considered robust if the results are fairly constant, even when the amount and/or selection of training data points are varied. To investigate this property, we first created a full segmentation by classifying all pixels of an image using a model that had been trained on a large training data set (100% of available expert-annotated data points). The result is considered the “reference prediction”. Then, we sampled training data subsets of varying sizes (50, 25, 10 and 5% of the training data, respectively) to train a model and predict the same image. We determined robustness by directly comparing this prediction with the reference prediction and calculating recall for each class. Results are visualized using violin plots as described above and the number of trials is again 500.

**Figure 4:**
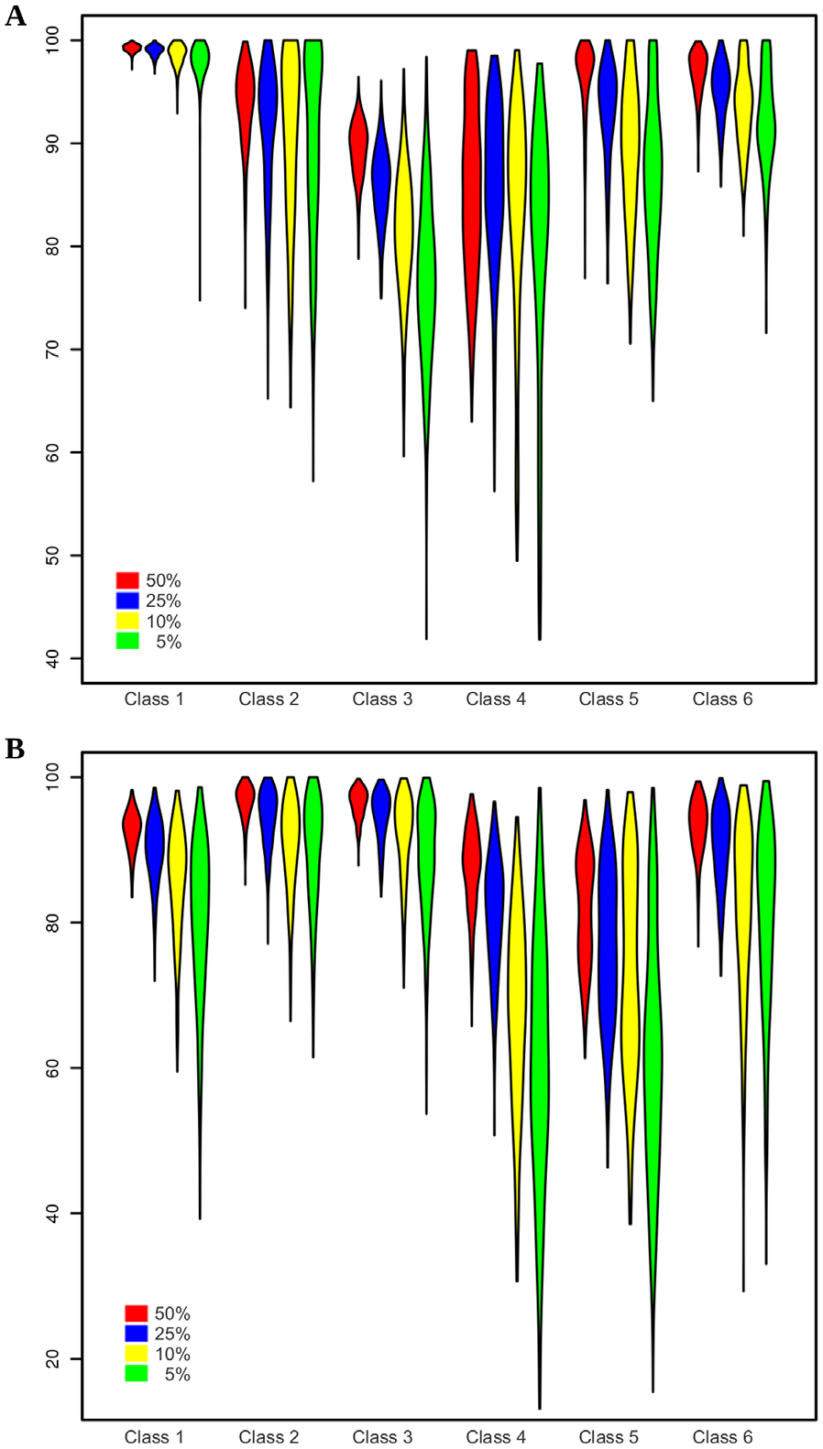
Cross-validation results of robustness analysis. Violin plots describing the robustness evaluated on the MIMS images of the cochlea (A) and brain (B), Figures 2A and 2E, respectively. For each class, recall was calculated by comparing the reference prediction (using all training data) with predictions using 50 (red), 25 (blue), 10 (yellow) or 5 (green) percent of randomly sampled training data. N = 500 in all cases.

Figure 4A shows that even with a small amount of training data, good classification results can be obtained for the cochlea image (Figure 2A) using the pixel-sampling scheme. An average recall greater than 75% can be achieved for all classes using just 5% of the training data. Furthermore, all classes reach an average recall of 80% or more using just 10% of the training data. Since the result does not heavily depend on training data size, this indicates the robustness of our SVM approach on the cochlea image. Figure 4B illustrates that satisfactory accuracy is obtained for all classes on the brain image (Figure 2E) with just 25% of the training data using the same pixel-sampling scheme. However, classes 4 and 5 clearly show a significant decrease in recall with a reduced fraction of training data. This result confirms the inferior class definitions already observed in the recall and precision analysis, again due to a significantly higher feature similarity between those classes and the total number of pixels defining these two classes being lower.

#### Homogeneity Analysis

**Figure 5:**
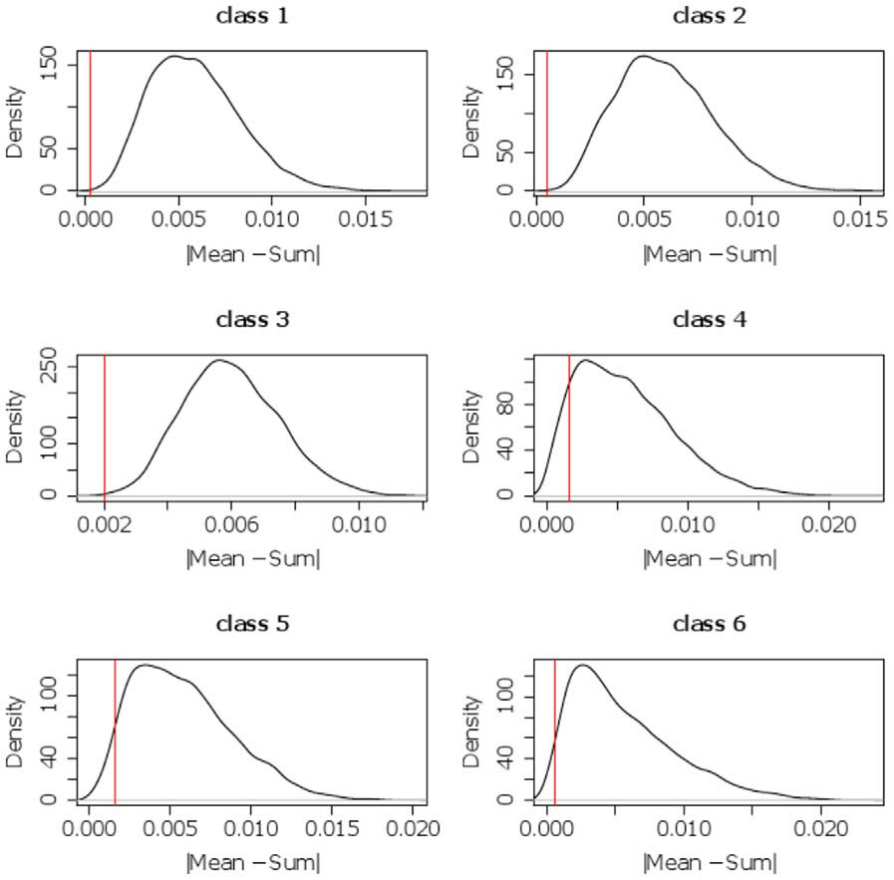
Null distributions of the homogeneity statistic. For each class, the approximated null distribution of the homogeneity statistic was calculated by random placement of predicted ROIs. Each distribution is displayed as histogram, smoothed with kernel density estimation. The true test statistic of each class was calculated based on the predicted ROIs and is indicated by a vertical bar within each plot. N = 10000.

The general aim of MIMS analysis is to compare a particular feature of interest—typically a ratio of masses (such as the ^12^C^15^N/^12^C^14^N ratio) in a tracer experiment—among the defined classes. If the algorithm has detected well-defined ROIs, all pixels belonging to one class are expected to be “homogeneous” with respect to the feature of interest. Homogeneity in this case refers to the ratios coming from a single population, irrespective of what that population looks like. In order to assess the homogeneity of class C with respect to the ratio of isotopic masses m_1_ and m_2_, we employ the statistic: h_C_ = |*Mean*−*Sum*|. *Mean* is calculated as the mean of all ratio image pixels *i* in class *C*, i.e.: *Mean* = (1/N)Σ(m_1,i_/m_2,i_). *Sum* is the result of the division of the sums of the individual mass image pixels m_1,i_ and m_2,i_ or *Sum* = (Σm_1,i_)/(Σm_2,i_). *Sum* can be thought of as “collapsing” the set of measurements of the ratio in *C* down to a single measurement of the isotopic ratio. For a given class, it can be shown that any gross discrepancy between *Mean* and *Sum* indicates that the ratios are distributed among at least 2 populations and thus are not homogeneous. P-values can be derived to estimate the significance of the results. We calculated the statistic h_C_ for each class on the brain image (Figure 2E) and tested it against the null-hypothesis that the ROIs had been randomly defined, that is, without any reference to the feature of interest. The null-distribution for each class was created by randomly translating each ROI of the respective class and calculating h_C_ after the full set of ROIs had been shifted. This procedure was repeated 10000 times for each class, and the resulting distributions are shown in Figure 5. By translating the ROIs, number and shapes of the original ROIs are maintained. This ensures that the random statistic is only affected by the positions of the ROIs and not, for instance, by total pixel number.

P-values are associated with the probability that a value at least as extreme as the one observed occurred only by chance and is not related to any homogeneous structure captured by the ROIs. Low p-values thus indicate that the corresponding ratio distribution is highly non-random and ROIs indeed cover a homogeneous set of pixels. In Figure 2E, the region of low counts on the right of the image, visible by the shadow on the HSI image, was excluded from the analysis to avoid any bias due to high noise in this region. Table 1 shows p-values for the 6 classes of the mouse brain image. P-values for classes 1, 2 and 3 are nominally zero. Thus, these results are highly significant and the ROIs can be assumed to be homogeneous with respect to the ^12^C^15^N/^12^C^14^N ratios. Figure 5 shows the null distribution for each class together with the homogeneity statistics calculated from the predicted ROIs of the respective classes. Classes 5 and 6 show slightly lower significance, and class 4 fails to pass the 5% significance level (indicating some inhomogeneity). This is in accordance with the previous results, which indicated that the SVM performance for these classes was inferior compared to the other classes. Users can also perform this test to validate their class definitions and final results. An output such as that shown in Figure 5 might lead the user to modify model training by either reconsidering training data selection or adjusting the number of defined classes. In summary, this analysis indicates a high degree of homogeneity within the majority of predicted classes and thus demonstrates the usefulness of our segmentation approach for biological interpretation of MIMS images.

**Table 1:** P-values of the homogeneity statistic h_c_ for each class of the mouse brain image (Figure 2E).

	p-value
**class 1**	0
**class 2**	0
**class 3**	0.0005
**class 4**	0.105
**class 5**	0.043
**class 6**	0.026

P-values were derived from a null-distribution by repeated random-assignments of ROIs. Nominal p-values of 0 occur if not a single random-sample achieved a better statistic than the observed one. Statistics for all classes, except for class 4, are significant at the 5% level. N = 10000.

#### Cross-Segmentation

**Figure 6:**
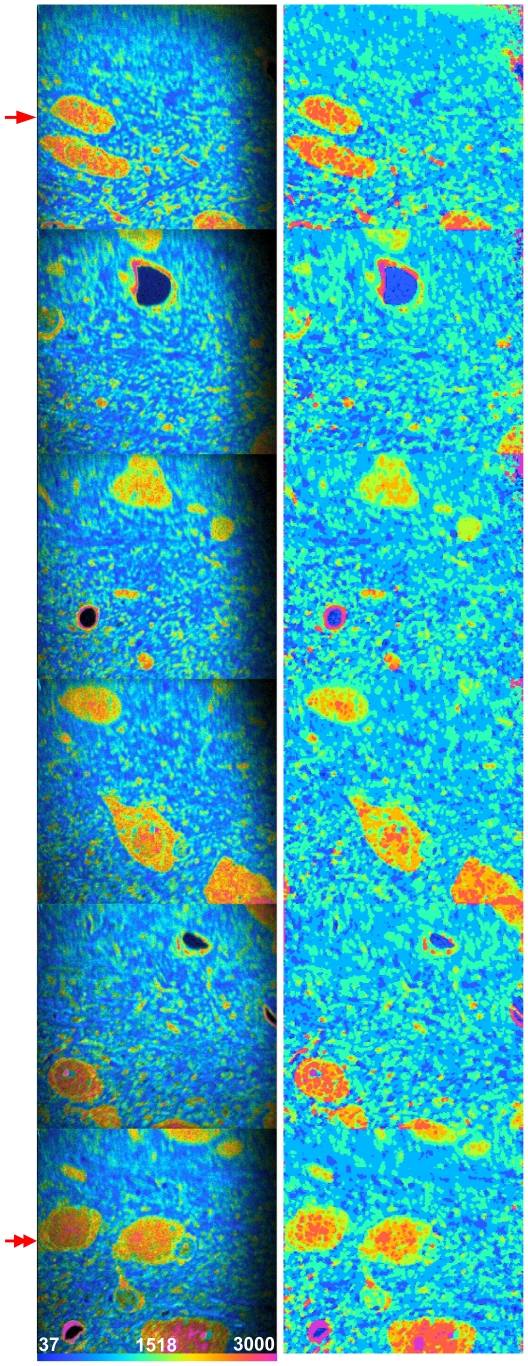
Cross-segmentation of consecutive brain sections. Example of segmentation of a series of images with the ^12^C^15^N/^12^C^14^N HSI shown in the left column and the full segmentation result shown in the right column; six fields total, 8 expert defined classes. The image from Figure 2E is at the top left (arrow). SVM trained on bottom left image (double arrow). These images were part of an acquisition of 37 images, 50×50 µm, 256×256 pixels, acquisition time 11 minutes.

In the validation steps described in the preceding sections, a dedicated SVM model had been trained for each image to be segmented. In many MIMS experiments, however, multiple images are generated under very similar experimental conditions (e.g., the acquisition of consecutive images on the same tissue section). Training models on each of them would hamper streamlined data analysis. To overcome this limitation, we evaluated the performance of an SVM model trained on a single image from a set of related samples and applied it to the complete set of images. Let F^X^ denote the model trained on image X and F^X^(Y) the segmentation result of applying model F^X^ to image Y. In case X = Y, this is referred to as “direct segmentation”. Otherwise, this is referred to as “cross-segmentation” of image Y by X. The performance of cross segmentation is assessed by pixel-wise comparison of the predicted and the expert annotated classes. In terms of robustness, the predicted image of cross segmentation was compared to the result of a direct segmentation (i.e., reference segmentation). Figure 6 illustrates the algorithm's ability to cross-segment a number of images from a data set based on training from a single image. The ^12^C^15^N/^12^C^14^N HSI images of six adjacent MIMS images are shown on the left and their full segmentation if shown on the right. The image at the top left of the figure (arrow) is the same as shown in Figure 2E, however the training data was taken from the bottom left image (double arrow).

**Figure 7:**
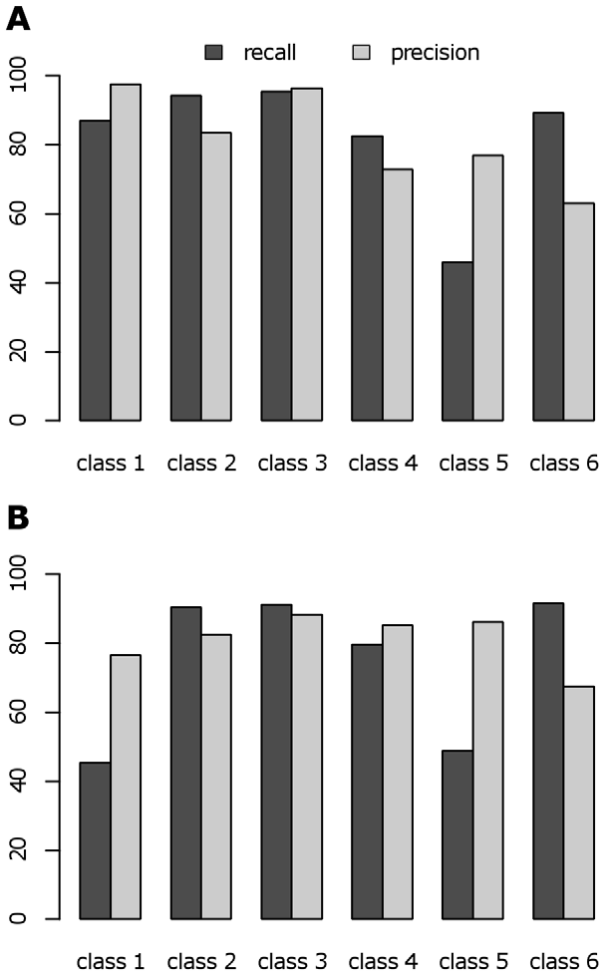
Cross-segmentation performance. Recall and precision values for each class of the cross-segmentation of the brain image in Figure 2E using a model trained an image from Figure 6 (bottom left image, double arrow). (A) The predictive performance of the cross-segmentation was evaluated on the expert-annotated data. (B) Comparison of direct- versus cross-segmentation based on the complete image segmentation.

First, we investigated the ability of a cross-segmentation to reproduce the expert's annotations on the original brain image (Figure 2E) using a model trained on the new image. Training ROIs were defined for 8 classes, 6 of which were equivalent to the 6 classes of the reference image. The two additional classes were defined to cover data points with extremely low and high ^12^C^15^N/^12^C^14^N ratios. This is a typical scenario where the SVM should be given training data that is as general as possible and spans the minimum ratio value (equivalent to the natural ratio) to the maximum value contained in the image series. Also, in contrast to the previous evaluations, the complete set of annotated ROIs was predicted and evaluated. Figure 7A shows the classification results. Cross-segmentation resulted in a recall over 80% in 5 of 6 classes and precision of over 70% in five of six classes. Lower recall and precision values in classes 5 and 6 might be due to the similarity of those classes, the difference between which could not be sufficiently learned on the other image.

To further examine the algorithm's usefulness, we compared the result from a cross-segmentation to that of a direct segmentation. ROIs were chosen by the expert on both brain images (Figure 2E and Figure 6 double arrow), and two SVMs were trained using those ROIs. We then compared the full segmentation of the image 2E using the SVM trained on image 2E (direct segmentation) with the full segmentation of image 2E using the SVM trained on the new image (cross-segmentation). Unlike the previous tests of recall and precision, all expert ROIs were used for training. Getting sufficiently equivalent results from both approaches would justify the use of cross-segmentation over having to train a dedicated model for each image. We show in Figure 7B the two results again in terms of recall and precision. Precision values are well above 70% in most cases, which demonstrates a satisfying confidence in the predicted classes. The recall of the algorithm is above 80% in most cases, however it is clearly impaired within two classes. This might be due to the greater richness of the cross-segmentation model, which contains a background class and a class representing the highest ratio values. Thus the model contains two classes more than actually exist on the target image, and thus simply increases the chance of false positive hits. This emphasizes that, for cross-segmentation to work well, it is crucial to define classes that are sufficiently distinct and consistent across images. It is the case that for most of the classes, this is feasible and thus makes cross-segmentation possible under carefully controlled analytical conditions. It should also be noted that the inclusion of a mass image in addition to the ratio image in the feature space can cause poor results when performing a cross-segmentation. This is due to overfitting, most likely caused by changes in the mass images that are canceled out in the ratio image (data not shown). This is the reason only the ^12^C^15^N/^12^C^14^N ratio image was given to the SVM for the brain images as previously stated.

## Design and Implementation

### Implementation

We used the libSVM package [21] to integrate the SVM algorithm into OpenMIMS, an ImageJ plugin for the comprehensive analysis of MIMS images (available at http://www.nrims.hms.harvard.edu/NRIMS_ImageJ.php). ImageJ is written in the platform-independent Java language and thus can be run on any operating system that provides the Java runtime environment (http://www.java.com). In addition to the default grid search (provided by libSVM), we implemented a Nelder-Mead simplex search [20] for tuning parameters of the SVM during training. Compared to the exhaustive grid search, this directed search method provides a significant improvement in terms of speed while showing equivalent performance in general [22]. The time required to train the SVM was reduced by a factor of 10–20 because the number of times the SVM must be retrained to perform cross validation is reduced by the same factor. All tests presented were performed using a radial basis function kernel and libSVM's standard one-to-one method of multi-class classification (Text S1).

### Design

The graphical user interface allows the user to define classes of interest and select training data, and provides access to the setup of the SVM. This includes choosing the feature space and the kernel type. Furthermore, functions are provided to automatically derive ROIs from the segmented image, which are subsequently available for comprehensive analysis by the OpenMIMS tool. For every step of the segmentation procedure, the user can save the current state, including defined classes and training data, the trained model and its configuration, and any prediction made by that model, along with the derived ROIs. The option of restoring a particular state at a later time is useful in the continuation of analysis and especially in the case where a previously trained model can be used to segment new data (cross segmentation).

## Availability and Future Directions

### Availability

This algorithm can be accessed on line at http://www.nrims.hms.harvard.edu/NRIMS_ImageJ.php. The source code, user and developer documentation, and several example data files are also available.

### Future Directions

**Figure 8:**
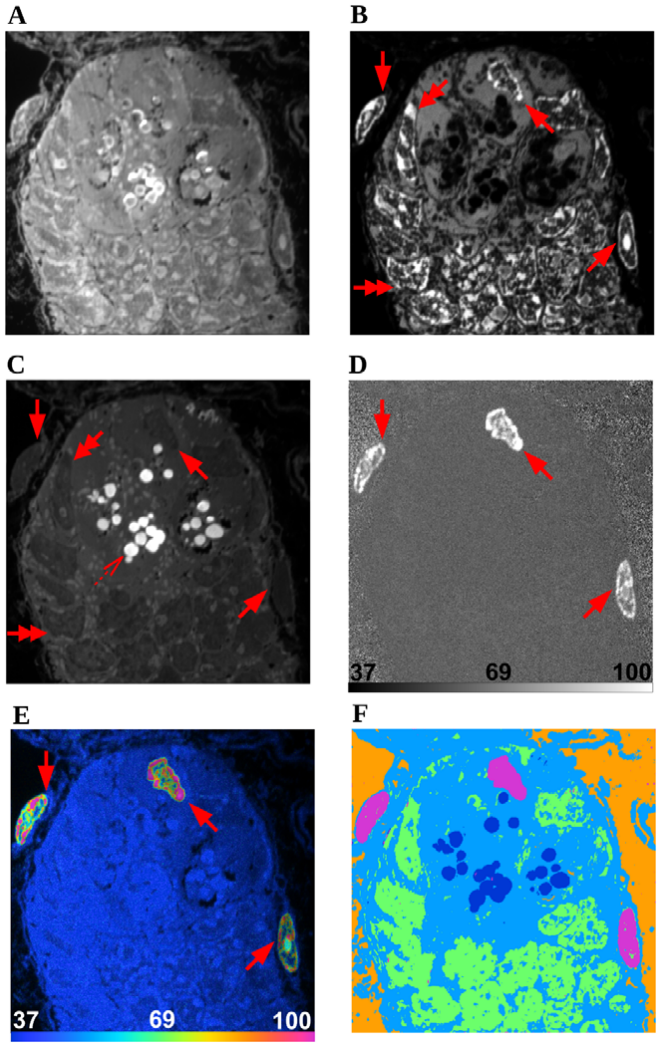
Segmentation of higher channel data. A MIMS image of a mouse intestinal crypt [23] showing ^15^N-thymidine labeled nuclei (solid arrows), unlabeled nuclei (double arrows) and sulfur-containing granules (outlined arrow). The images are ^12^C^14^N (A), ^31^P (B), ^32^S (C), ^12^C^15^N/^12^C^14^N (D), ^12^C^15^N/^12^C^14^N HSI (E). Scale bars in (D) and (E) range from the natural ratio to a value that clearly delineates the borders of labeled nuclei (times 10000). The resulting segmentation is shown in (F). Field 30×30 µm, 512×512 pixels, acquisition time 849 minutes.

An SVM approach was chosen for its general applicability, so that it has the ability to work with data of varying dimensions. The algorithm can be applied to images with a higher number of channels (i.e. mass or ratio images) than the ones used in this study. Figure 8 shows initial results of using the algorithm to segment a crypt of the small intestine [23] with regions of high ^15^N label, as well as areas with endogenous phosphorous and sulfur. In contrast to the previously shown MIMS images of mouse cochlea and brain (Figures 1,2,6), which quantitate the level of protein turnover, this data comes from an experiment where newly synthesized DNA was labeled via the introduction of ^15^N-thymidine. The algorithm clearly reveals labeled nuclei (solid arrows), unlabeled nuclei (double arrows and visible in the P and S channels) and sulfur-containing granules (outlined arrow). Because the signal of different masses can vary by orders of magnitude, some asymmetry may need to be introduced into the algorithm to increase accuracy (e.g., a per-image radius for the computation of neighborhood statistics).

**Figure 9:**
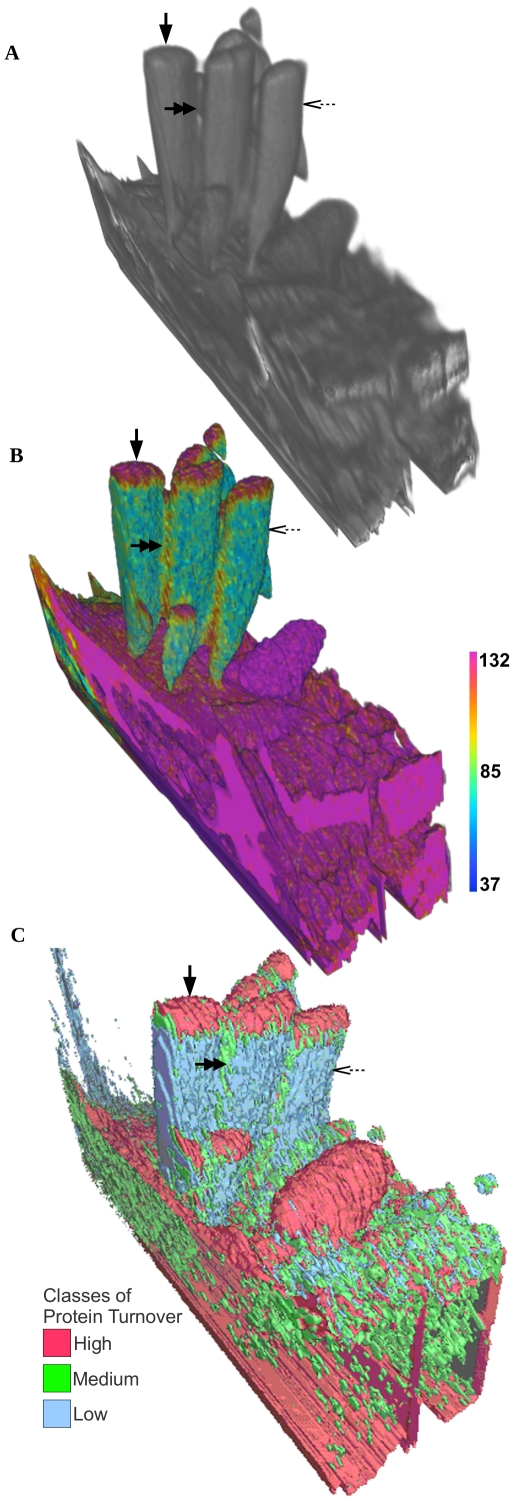
Segmentation of Volumes. MIMS image of mouse stereocilia [24] from an 8×8×2-µm volume in 256×256×90 voxels (acquisition time 1966 minutes). Renderings of ^12^C^14^N (A), ^12^C^15^N/^12^C^14^N HSI (B) both rendered in ImageVis3D [25]. The scale bar in (B) ranges from the natural ratio to the value in the ^15^N-enriched chow, which corresponds to the maximum ratio that could be reached in newly synthesized protein (multiplied by 10000). Regions of high turnover (solid arrow), medium turnover (double arrow), and low turnover (outlined arrow) are clearly visible in (B). The resulting segmentation (C) is rendered in Seg3D [26] and these same classes of high, medium, and low turnover are shown colored as red, green, and blue respectively.

The lack of positional information in the feature space allows the same model to apply to sequences of images. This “cross-segmentation” approach has been used to segment multiple images of consecutive tissue slices (Figure 6). This can also be applied to images that extend in the Z direction. The application of this method to an 8×8×2 µm volume of MIMS images from a mouse inner ear stereocilia [24] is shown in Figure 9. The SVM was trained on the ^12^C^15^N/^12^C^14^N volume (the inclusion of only the ratio data is for the same reasons outlined in the previous section *Cross-Segmentation* and Figure 6) and given 3 classes of high, medium, and low ^12^C^15^N/^12^C^14^N ratio (or protein turnover) that were defined by the expert on a single slice of the volume. The algorithm successfully found the regions of high turnover (solid arrow), medium turnover (double arrow), and low turnover (outlined arrow) visible in the ^12^C^15^N/^12^C^14^N HSI in Figure 9B. The complete segmentation of these 3 classes is shown in Figure 9C. Renderings of the data were made using ImageVis3D [25] and Seg3D [26]. In the future, methods able to split regions that represent distinct structures but may be covered by a single segmented, connected volume would further improve the application.

## Supporting Information

Text S1 **A short discussion of SVMs, the Nelder-Mead algorithm, and violin plots.**
(DOC)

Figure S1 **SVM Classification.** A schematic showing 2 classes of data points (gray and black circles) and 3 separating hyperplanes (H_1_–H_3_) in 2 dimensions. H_1_ (red line) does not separate the data. Both H_2_ (green line) and H_3_ (blue line) separate the data; however the margin (black lines) of H_3_ being the largest possible margin, H_3_ is the separating hyperplane found by the SVM. The points closest to H_3_ are the eponymous “support vectors”.
(TIF)

Figure S2 **Linearly Separable Data.** A representation of the mapping of data from a space where the classes are not linearly separable to one where they are. This mapping *Φ* is related to a given kernel function *k* by *k(* ***x_i_, x_j_*** *) = Φ(* ***x_i_*** *)·Φ(* ***x_j_*** *)* where ***x_i_*** and ***x_j_*** are data points.
(TIF)

Figure S3 **Violin Plots.** A reproduction of a single violin from Figure 3A of the manuscript to illustrate a violin plot. Gray bars have been added to show the range of measured values of a sample (vertical grey bar) and the estimated relative probability of obtaining a given value from one sample (horizontal grey bar, value = 90%).
(TIF)

Figure S4 **Brain Image Acquisition.** A light microscopy image of a sagittal section of embedded mouse hippocampus showing the approximate position of the 2 brain images used in the manuscript. Also shown is the path of acquisition of the dataset these 2 images were taken from.
(TIF)

## References

[1] Lechene C, Hillion F, McMahon G, Benson D, Kleinfeld AM (2006). High-resolution quantitative imaging of mammalian and bacterial cells using stable isotope mass spectrometry.. Journal of Biology.

[2] Lechene C, Luyten Y, McMahon G, Distel D (2007). Quantitative imaging of nitrogen fixation by individual bacteria within animal cells.. Science.

[3] McMahon G, Francois Saint-Cyr H, Unkefer CJ, Lechene C (2006). CN^−^ Secondary Ions Form by Recombination as Demonstrated Using Multi-Isotope Mass Spectrometry of ^13^C- and ^15^N-labeled Polyglycine.. Journal of the American Society for Mass Spectrometry.

[4] Pal NR, Pal SK (1993). A review on image segmentation techniques.. Pattern Recognition.

[5] Székely G, Gerig G (2000). Model-based segmentation of radiological images.. KI Künstliche Intelligenz.

[6] Cortes C, Vapnik V (1995). Support-Vector Networks.. Machine Learning.

[7] Boser BE, Guyon IM, Vapnik VN, Haussler D (1992). A training algorithm for optimal margin classifiers..

[8] Kreßel U, Schölkopf B, Burges CJC, Smola AJ (1999). Pairwise Classification and Support Vector Machines.. Advances in Kernel Methods — Support Vector Learning.

[9] Hsu C-W, Lin C-J (2002). A Comparison of Methods for Multiclass Support Vector Machines.. IEEE Transactions on Neural Networks.

[10] Brown MPS, Grundy WN, Lin D, Christianini N, Sugnet CW (2000). Knowledge-based analysis of microarray gene expression data by using support vector machines.. PNAS.

[11] Guyon I, Weston J, Barnhill S, Vapnik V (2002). Gene Selection for Cancer Classification using Support Vector Machines.. Machine Learning.

[12] Hua S, Sun Z (2001). A novel method of protein secondary structure prediction with high segment overlap measure: support vector machine approach.. J Mol Biol.

[13] El-Naqa I, Yang Y, Wernick MN (2002). A Support Vector Machine Approach for Detection of Microcalcifications.. IEEE Transactions on Medical Imaging.

[14] Zhang X, Lu X, Shi Q, Xu X, Leung HE (2006). Recursive SVM feature selection and sample classification for mass-spectrometry and microarray data.. BMC Bioinformatics.

[15] Burges C (1998). A Tutorial on Support Vector Machines for Pattern Recognition.. Data Mining and Knowledge Discovery.

[16] Cristianini N, Shawe-Taylor J (2000). An Introduction to Support Vector Machines and Other Kernel-based Learning Methods.

[17] Schölkopf B, Tsuda K, Vert JP (2004). Kernel Methods in Computational Biology.

[18] Frank E, Kessler MS, Filiou MD, Zhang Y, Maccarrone G (2009). Stable Isotope Metabolic Labeling with a Novel ^15^N-Enriched Bacteria Diet for Improved Proteomic Analyses of Mouse Models for Psychopathologies.. PLoS ONE.

[19] Zawadzki RJ, Fuller AR, Wiley DF, Hamann B, Choi SS (2007). Adaptation of a support vector machine algorithm for segmentation and visualization of retinal structures in volumetric optical coherence tomography data sets.. Journal of Biomedical Optics.

[20] Nelder JA, Mead R (1965). A Simplex Method for Function Minimization.. The Computer Journal.

[21] Chang CC, Lin CJ (2011). libSVM: a library for support vector machines.. ACM Transactions on Intelligent Systems and Technology.

[22] Cohen G, Ruch P, Hilario M, Russell I, Markov Z (2005). Model Selection for Support Vector Classifiers via Direct Simplex Search.. FLAIRS.

[23] Steinhauser M, Bailey A, Senyo S, Guillermier C, Perlstein T (in press). Multi-isotope imaging mass spectrometry quantifies stem cell division and metabolism.. Nature.

[24] Zhang D-S, Piazza V, Perrin B, Rzadzinska A, Poczatek JC (In press). Multi-istope imaging mass spectrometry reveals slow protein turnover in hair-cell stereocilia.. Nature.

[25] ImageVis3D: A Real-time Volume Rendering Tool for Large Data. Scientific Computing and Imaging Institute (SCI).. http://www.imagevis3d.org.

[26] Seg3D: Volumetric Image Segmentation and Visualization.. http://www.seg3d.org.

