# Supplementary material for: Segmentation of Multi-Isotope Imaging Mass Spectrometry Data for Semi-Automatic Detection of Regions of Interest
Source: PLoS One. 2012 Feb 9;7(2):e30576. doi: 10.1371/journal.pone.0030576 (PMC3276494; doi:10.1371/journal.pone.0030576)
Supplement: Text S1 — A short discussion of SVMs, the Nelder-Mead algorithm, and violin plots. (DOC) [file pone.0030576.s001.doc]

**Supplemental Text S1:**

Below are brief and non-technical descriptions of three terms used in the manuscript: *SVM*, *Nelder-Mead*, and *violin plot*. For exact implementations, our code is available on our website: <http://www.nrims.hms.harvard.edu/NRIMS_ImageJ.php>. For more technical overviews of SVMs, see [15-17].

**SVM:**

SVMs (or support vector machines) are a widely used class of classification algorithms. If one has a set of data of dimension *d,* an SVM finds a *d-1* dimension *hyperplane* which separates the data into 2 classes. Here a *hyperplane* is the higher dimensional analog to the 2 dimensional plane in a 3 dimensional space. In general, there may be many hyperplanes that separate the data. Instead of finding any hyperplane, an SVM finds the solution to the optimization problem that is: which hyperplane has the largest margin? the margin being the distance between the hyperplane and the closest data points (see Supplemental Figure S2). These closest data points are the *support vectors*. If this separation is possible with no mis-classifications the data are termed *linearly separable*.

Often, however, the data are not linearly separable. One method to overcome this is to use a *soft margin* by introducing a penalty parameter for misclassified data points [6]. This parameter is denoted as *C* in libSVM [21].

Another very important method is the transformation of the data from a space where it is not linearly separable to a space where it is by using what is termed a *kernel function* [7]. Supplemental Figure S3 gives a representation of this transformation. Many different types of kernels have been used for various types of data. We have used the radial basis function (RBF) given by the equation *k(****x_i_, x_j_****) = exp(-γ||****x_i_*** *-* ***x_j_****||^2^ )* . Both *C* and *γ* are parameters for which optimum values need to be found to maximize the accuracy of the SVM.

When data contains more than 2 classes an SVM is constructed for each pair of classes [8]. For a given data point, the class with the most votes from this set of SVMs determines the final class membership. This method is commonly called *one-to-one*, is implemented in libSVM [21], and has been shown to perform well in general [9].

**Nelder-Mead:**

In order to train an SVM when using an RBF kernel, optimum values for the parameters *C* and γ must be found. Often this is done using a grid search to test every combination of *C* and γ over appropriate ranges. The testing of every parameter pair is somewhat computationally heavy, yet a new search must be performed for each new data set because we can make no assumptions that previous values will give good performance on new data sets (in most cases). Therefore we implemented a version of the Nelder-Mead [20] algorithm to speed up the search.

Nelder-Mead is a type of gradient descent algorithm that seeks the minimum of some function. In our case, we would like to maximize accuracy, which is the same as minimizing error. Here we describe the case of using Nelder-Mead in two dimensions, though it can be used to minimize a function with a domain of higher dimension.

In 2 dimensions, 3 points are chosen randomly over the space of possible values *C* and γ forming a triangle. These 3 points allow for an approximation of the gradient of the error function. Although there can be many variations in the implementation of the algorithm, basically at each step one of three situations can occur:

- one or more points of the triangle can contract giving a smaller triangle

- one or more points of the triangle can expand giving a larger triangle

- one of the points of the triangle can be reflected across the opposite edge

The behavior of the algorithm can be visualized as this triangle of points walking down the gradient of the error function and eventually contracting around a minimum. It should be clear that it is not guaranteed to find the global minimum, but in our tests it performed very well. Note that the increase in speed is due to the reduction in the number of points for which the SVM needs to be trained and the error calculated. In our testing this reduction has been by a factor of 10-20.

**Violin Plot:**

A violin plot is a representation of numerical data. Given a sample, the probability density function of the underlying distribution is estimated from the histogram of the data. This estimate is then used to create a "violin" where the width at a given level of the Y-axis is proportional to the probability that a measurement takes a specific value. This allows the viewer to make quick comparisons of distributions between many samples when visualizing many highly non-normal distributions. A part of Figure 3A from the manuscript is included in Supplemental Figure S4 as an illustration.
