# Supplementary figures and images for: Segmentation of Multi-Isotope Imaging Mass Spectrometry Data for Semi-Automatic Detection of Regions of Interest
Source: PLoS One. 2012 Feb 9;7(2):e30576. doi: 10.1371/journal.pone.0030576 (PMC3276494; doi:10.1371/journal.pone.0030576)

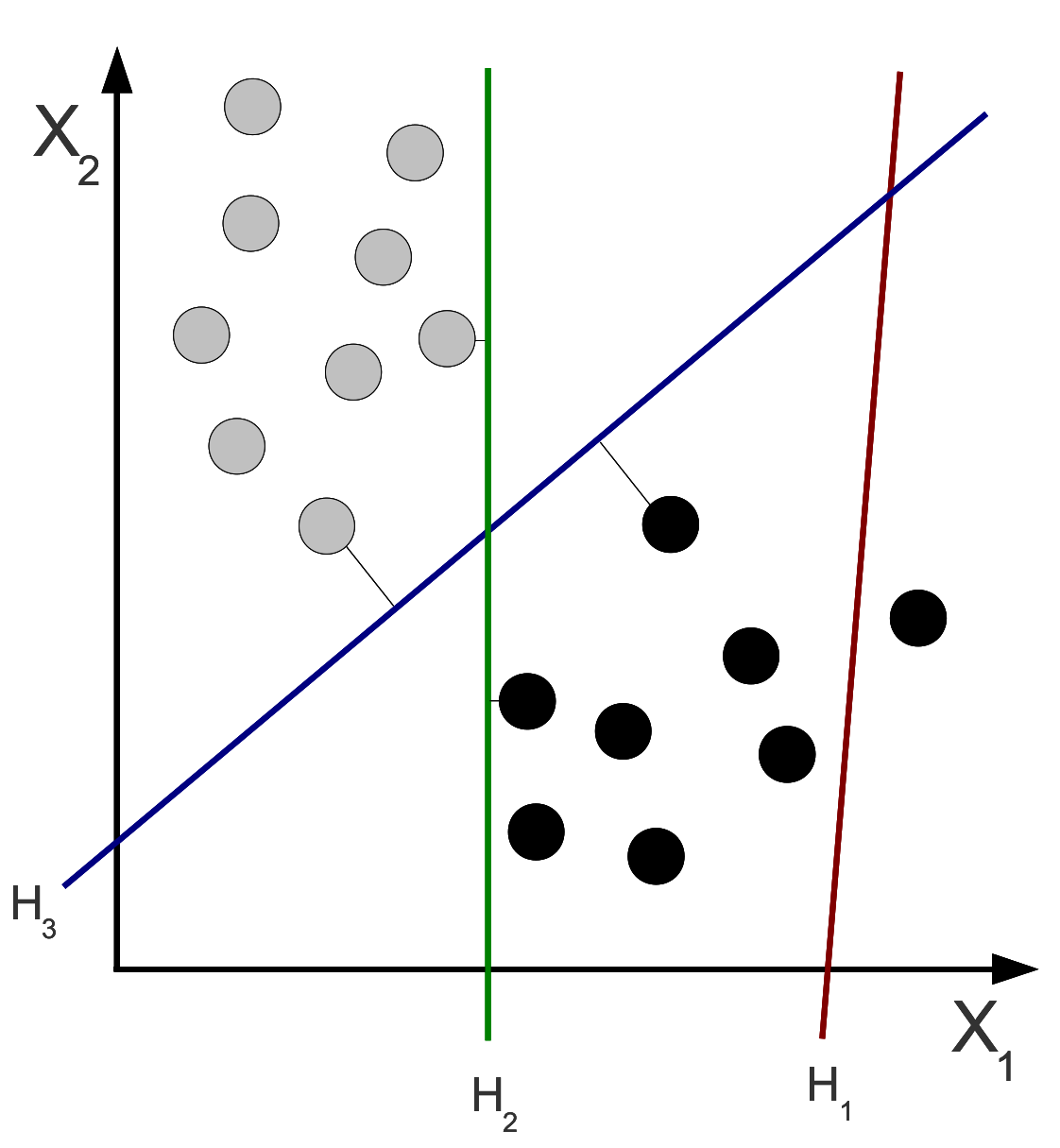

Supplement: Figure S1 — SVM Classification. A schematic showing 2 classes of data points (gray and black circles) and 3 separating hyperplanes (H1–H3) in 2 dimensions. H1 (red line) does not separate the data. Both H2 (green line) and H3 (blue line) separate the data; however the margin (black lines) of H3 being the largest possible margin, H3 is the separating hyperplane found by the SVM. The points closest to H3 are the eponymous “support vectors”. (TIF) [file pone.0030576.s002.tif]

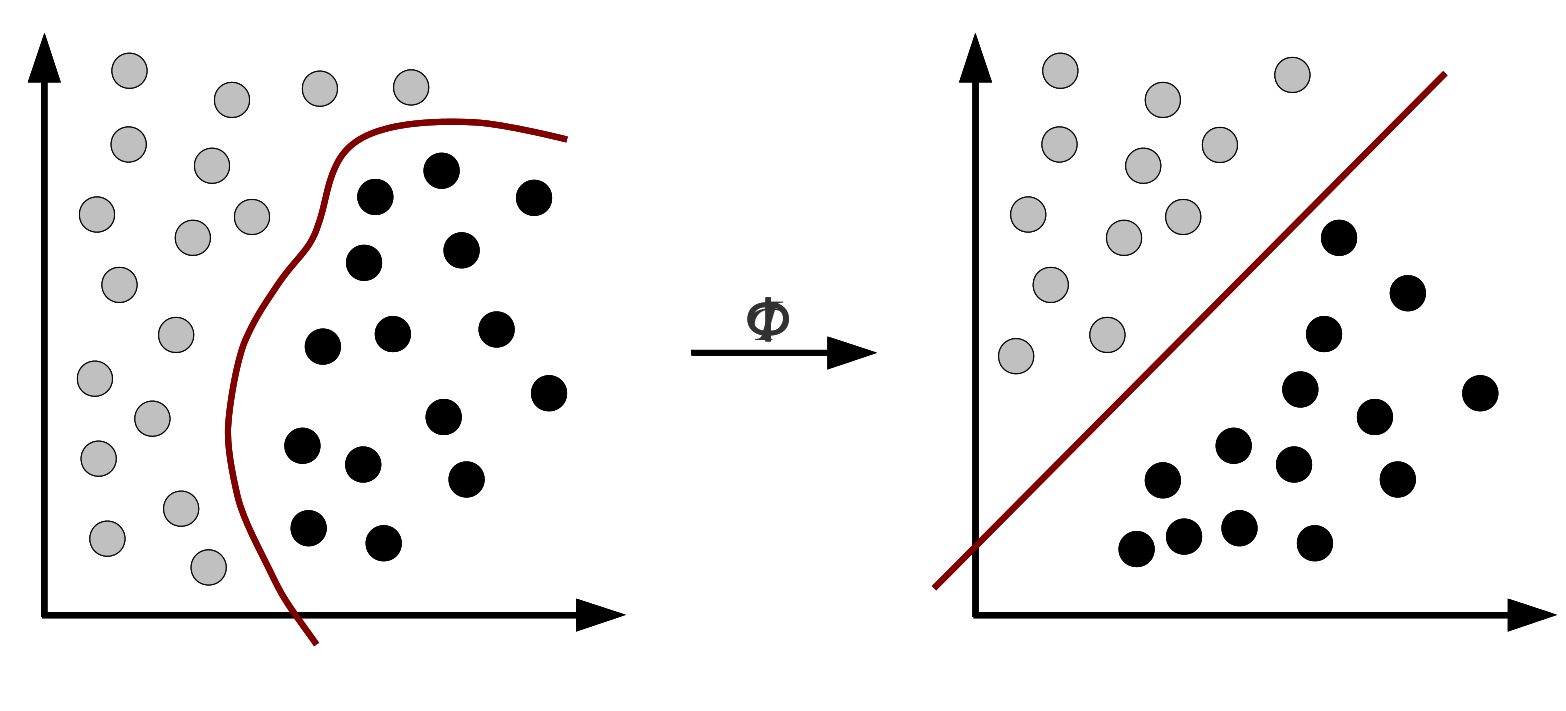

Supplement: Figure S2 — Linearly Separable Data. A representation of the mapping of data from a space where the classes are not linearly separable to one where they are. This mapping Φ is related to a given kernel function k by k( xi, xj ) = Φ( xi )·Φ( xj ) where xi and xj are data points. (TIF) [file pone.0030576.s003.tif]

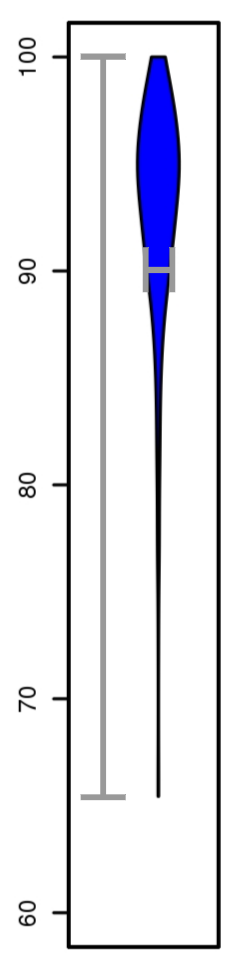

Supplement: Figure S3 — Violin Plots. A reproduction of a single violin from Figure 3A of the manuscript to illustrate a violin plot. Gray bars have been added to show the range of measured values of a sample (vertical grey bar) and the estimated relative probability of obtaining a given value from one sample (horizontal grey bar, value = 90%). (TIF) [file pone.0030576.s004.tif]

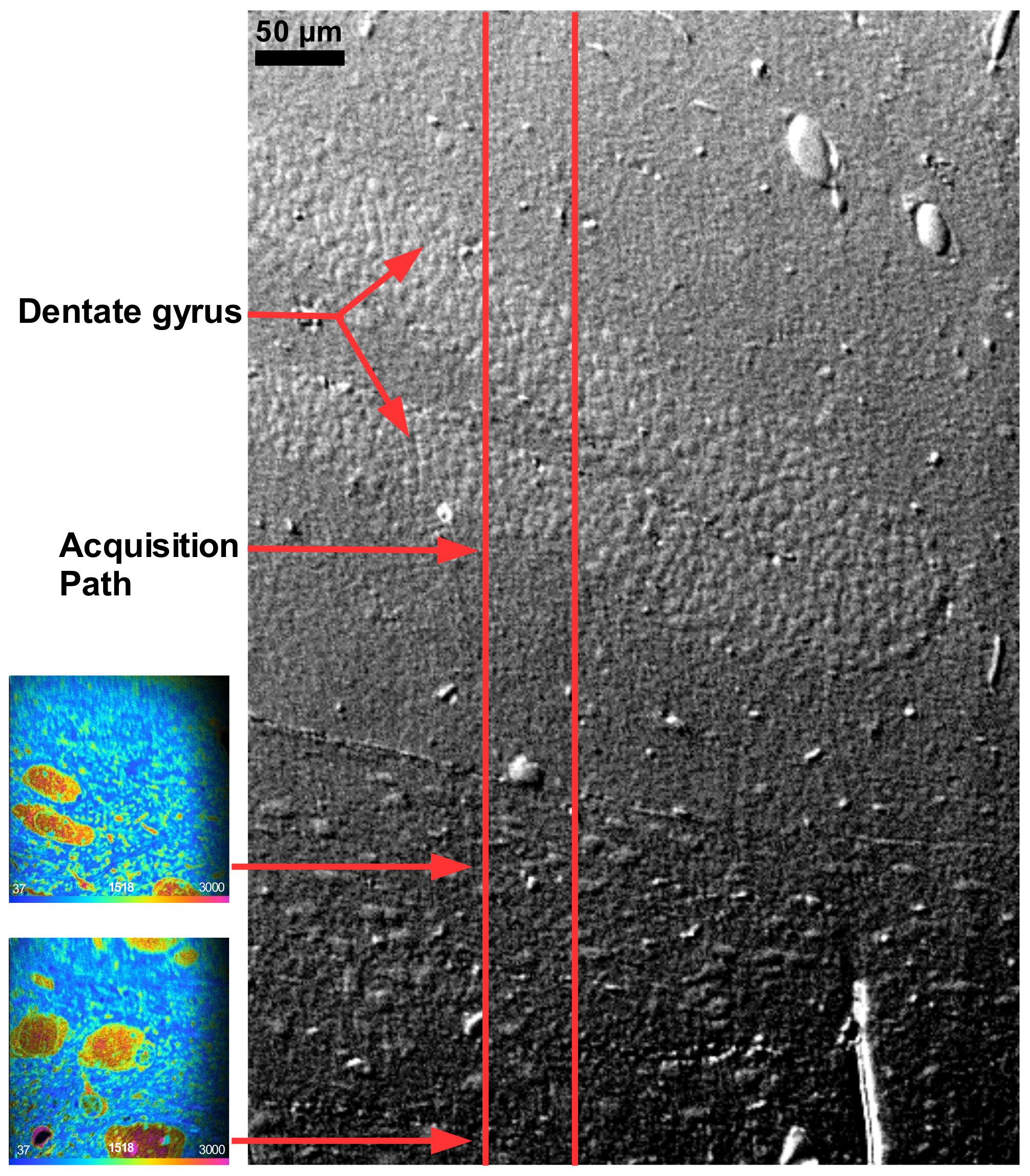

Supplement: Figure S4 — Brain Image Acquisition. A light microscopy image of a sagittal section of embedded mouse hippocampus showing the approximate position of the 2 brain images used in the manuscript. Also shown is the path of acquisition of the dataset these 2 images were taken from. (TIF) [file pone.0030576.s005.tif]
